# Effect of Internal Support on the Tensile Properties and Fracture Mode of 304 Stainless Steel Thin-Walled Tubes

**DOI:** 10.3390/ma14010172

**Published:** 2020-12-31

**Authors:** Yue Gao, Fei Shao, Pengxian Fan, Qian Xu, Xingkun Xie

**Affiliations:** 1 Field Engineering College, Army Engineering University of PLA, Nanjing 210007, China; q1013385211@sina.com (Y.G.); q1058427910@sina.com (Q.X.); jfxie2020@sina.com (X.X.); 2 Defense Engineering College, Army Engineering University of PLA, Nanjing 210007, China

**Keywords:** internal support, 304 stainless steel, thin-walled tube, tensile property, fracture mode

## Abstract

Steel–tube composite structures contain multiple tubular components under tension. The enhancement of the mechanical properties of tubes under ultimate operating conditions is crucial for improving structural safety. In this study, 110 pieces of 304 stainless steel thin-walled tubes (SSTWTs) under five internal support conditions are investigated. The ultimate tensile strength, ultimate extension, and fracture energy of different groups of specimens are measured to understand the variation mechanism of fracture modes. The elastic modulus of tube filler is treated as a variable to establish a uniaxial tensile fracture matrix of 304 SSTWTs with different tube fillers and loading rates. The results demonstrate that flexible tube fillers can effectively limit the lateral necking of 304 SSTWTs. Under the middle fracture mode, the maximum increments in the ultimate strength, extension, and fracture energy of tubes are 10.81%, 24.56%, and 35.94%, respectively. Furthermore, as the support rigidity increases, the ultimate strength exhibits an overall increasing trend, while the extension and fracture energy initially increase and then decrease. Overall, this study provides a novel route for enhancing the performance of steel–tube composite structures under ultimate loading conditions, which is of great significance for improving the safety of the structural design and reducing the engineering construction cost.

## 1. Introduction

Tubular components are extensively applied in aerospace, offshore platforms, power transmission facilities, energy and chemical industries, and transportation [1,2,3,4,5,6,7]. In the 2020 Global Engineering Frontier Survey conducted by the Chinese Academy of Engineering, “Design and construction technology of prefabricated steel–tube composite bridge” has been recognized as a candidate direction for cutting-edge issues in engineering development. Owing to the increasing applications of tubular components in the engineering field, there is an urgent demand for their higher technical indicators. In composite structures, tension is the common state of stress for steel tubes. During the service process under extreme conditions such as earthquakes, explosions, impacts, ice, and snow disasters, structural tubes suffer excessive tensile loads, which causes strength failure. The failure process is generally a complex deformation process. Hence, the study of the ultimate mechanical properties of tubes under extreme conditions has become a key issue for engineering researchers.

The exploration of the tensile properties of hollow tubes is a relatively mature field. Cai et al. [8] quantitatively analyzed the surface defects in an orange peel of 6063 aluminum alloy spinning tubes during the stretching process using tensile tests. The results revealed that with the increase in strain, the surface roughness initially increased and then decreased, and the coarsening rate dropped exponentially. Khandelwal et al. [9] investigated the influence of temperature, sample position, and ingot melting on the mechanical properties of Zr-2.5Nb alloy pressure tubes, and they suggested that the four-fold smelting method improved the fracture toughness of materials under different manufacturing routes. Guo et al. [10] explored the effects of annealing on the microstructure evolution and tensile properties of skew-rolled Ti-6Al-3Nb-2Zr-1Mo alloy tubes. It was found that the yield strength, ultimate tensile strength, and extension rate increased with the increase in annealing temperature. Chen et al. [11] studied the crack propagation of a 1Cr18Ni9Ti tube during tensile fracture and derived an approximate equation for real tensile stress at any moment during the uniform deformation process. Furthermore, using finite-element analysis, the stress and strain distributions during the necking process were analyzed. Yang et al. [12] investigated the structural degradation and tensile properties of 12Cr1MoV steel tubes after long-term service and compared them with those of nonserviced steel tubes using tensile fracture analysis under high temperature and room temperature. After long-term service, the room-temperature yield strength, high-temperature yield strength, and tensile strength of the steel tube were significantly reduced. Qu et al. [13] studied the axial tensile properties of annealed and martensitic M5 zirconium alloy cladding tubes at room temperature by static tensile tests and visualized the static tensile fracture by scanning electron microscopy (SEM). The martensitic M5 zirconium alloy tube exhibited higher strength than the annealed M5 zirconium alloy tube, but its plasticity and toughness were lower. The axial tensile fracture of both kinds of M5 zirconium alloy tubes exhibited dimple morphology, and the fracture type exhibited a ductile fracture.

Owing to its unique hollow structure, a tube undergoes more evident lateral necking when it is stretched longitudinally. The lateral necking in the middle part of the tube intensifies the stress concentration at the necking part, thereby accelerating the fracture in this part of the tube. Consequently, the mechanical properties of other parts of the tube are not utilized completely. The tube filler set in the tube limits the lateral necking of the middle part of the tube to a certain extent, which makes the entire tube section more uniformly deformed, thereby improving the overall mechanical performance of the tube. Hence, a clear understanding of the influence of internal support on the mechanical properties and fracture mode can serve as a useful guide for accurately describing and improving the mechanical performance of the tube.

The existing studies on tubes with an internal filling mainly focus on the ultimate bearing capacity [14,15,16], seismic performance [17,18,19], numerical modeling [20,21,22] of concrete-filled steel tubes (CFSTs) and their energy absorption characteristics [23,24,25,26], compression behavior [27,28,29], and crashworthiness analysis [30,31] of thin-walled tubes filled with foam metal. Most of the studies investigated the compression performance of tubes filled with a certain material. However, there are relatively few studies on the tensile properties of internal prefilled tubes. Some studies have explored the tensile properties of CFST members. In particular, Han et al. [32], Tao et al. [33], Xu et al. [34], Li et al. [35,36], and Pan et al. [37] have extensively examined various types of CFST members to reveal meaningful conclusions. Han et al. [32] investigated the force mechanism of 18 CFST specimens under axial tension and found that the tensile strength of CFST specimens increased by 11% due to the filling of concrete. Furthermore, a simple equation for predicting tensile strength was proposed. Zhou et al. [33] conducted an in-depth study on the mechanical behavior of squared concrete-filled steel tubes (SCFSTs) under axial tension and concluded that the tensile strength of SCFST was on average 5.2% higher than that of hollow tubes. Xu et al. [34] established an analysis model for the enhancement of strength and rigidity of a circular concrete-filled steel tube (CCFST) under axial tensile load. In this model, the performance of the interface between steel tubes and concrete was considered. Additionally, an analytical formula for the enhancement coefficient of strength and rigidity was established. Chen et al. [35] used reinforced CFST columns embedded with steel bars or steel angles under eccentric tension to propose simplified design equations for the elastic tensile stiffness and tension versus moment interaction relationship of reinforced CFSTs. Ye et al. [36] experimentally studied the mechanical behavior of concrete-filled stainless steel tubes (CFSSTs) subjected to eccentric tension. It was found that the effective combination of concrete filler and an external stainless steel tube increased the tensile strength by 5–10% compared to that of the corresponding hollow stainless steel tube. Pan et al. [37] used deformation coordination conditions to analyze the mechanical properties of CFSTs under axial tension and found that the presence of core concrete increased the longitudinal yield stress of the steel tube by nearly 10%. Li et al. [38] conducted eccentric tensile tests on concrete-filled double-skin steel tube (CFDST) specimens to conclude that the initial rigidity of the specimens was much higher than that of the bare steel tubes, and the tensile strength was significantly higher than that of the hollow steel tubes. Furthermore, and the ultimate tensile strength decreased with the increase in eccentricity.

Based on the previously reported tensile tests of various types of CFSTs, it can be concluded that concrete filler plays a significant role in improving the tensile strength of steel tubes. However, the ductility and fracture energy of steel tubes have been rarely investigated. The effect of internal support on the fracture mode of steel tubes has not been analyzed yet. In this study, to address these issues, fracture tests are conducted on 304 SSTWTs. The ultimate nominal tensile strength (UNTS), extension, fracture energy, and fracture mode of tubes with different tube fillers and loading rates are obtained and compared with the stretching results of empty tubes. Based on the above results, the critical loading rates for the switching of the fracture mode of 304 SSTWTs under different tube fillers are explored. Furthermore, the impact of flexible and rigid internal supports on the mechanical properties and fracture mode of tubes is examined. It is demonstrated that the use of polyurethane (PU) as a tube filler can significantly improve the comprehensive mechanical performance of the tube within a wide range of loading rates.

## 2. Materials and Methods

### 2.1. Sample Preparation

In this study, industrial 304 stainless steel (Zhejiang Yihong Stainless Steel Co., Ltd., Lishui City, China) (AISI/ASTM, 0Cr18Ni9 in China GB/T14976-2012) thin-walled tubes were used for investigation, the chemical composition of which is shown in Table 1.

The actual dimensions of circular tubes were $D_{0}=30.0$ mm, $d_{0}=25.7$ mm, and $S_{0}=188.1$ mm^2^. According to ISO 6892-1 Metallic Materials-Tensile testing and GB/T 228.1, the original gauge length of the specimen was $L_{0}=5.65\sqrt{S_{0}}=77.5$ mm. Hence, *L_0_* was set as 80 mm. The parallel length of the specimen was $L_{c}\geq L_{0}+D_{0}/2=95$ mm. Here, *L_c_* was set as 95 mm. According to the size of the fixture of the testing machine, the clamping length was $L_{h}=60$ mm. Based on the values of $L_{c}$ and $L_{h}$, the overall length of the specimen ($L_{t}$) was $L_{t}=L_{c}+2L_{h}=215$ mm.

Q235 (“Q235” (Quzhou Yuanli Metal Products Co., Ltd., Quzhou, China) is a steel grade in China, representing ordinary carbon structural steel with a yield strength of 235 MPa. “Q235” is equivalent to “A570” of the American ASTM standard) circular steel was selected as the plug head of the clamping end. The plug head was used to prevent the deformation of the tube due to the clamping of the testing machine. The plug only supported the inside part of the tube and did not bear tensile force in the longitudinal direction. The length of the plug head of the clamping end was *L_b_* = 60 mm.

**Figure 1:**
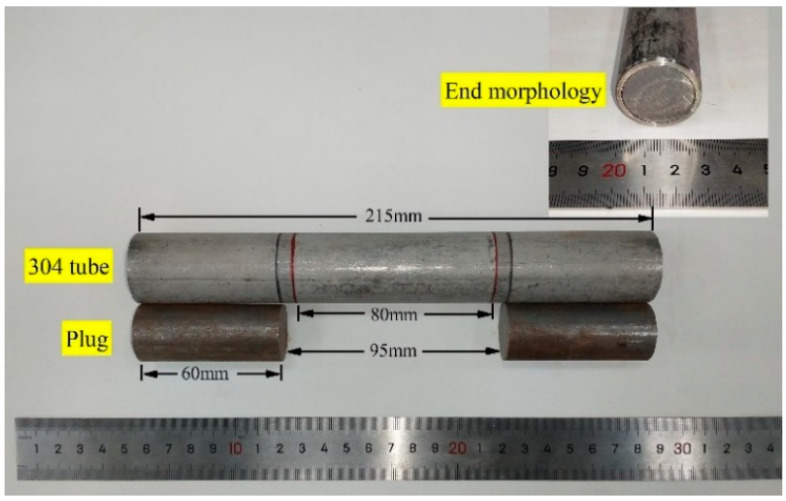
Standard specimen of a 304 SSTWT.

According to the size requirements, a standard specimen of a 304 SSTWT was fabricated. An image of this specimen is shown in Figure 1.

**Figure 2:**
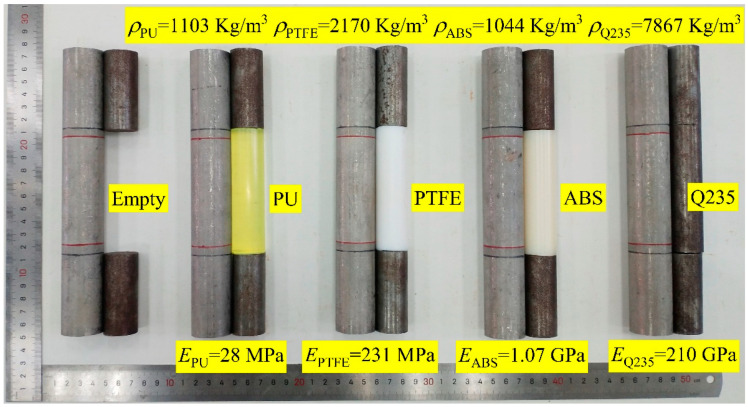
Internal support conditions.

There were five internal support working conditions: one included an empty tube, and the other four included filled tubes. The four supporting materials were polyurethane (PU), polytetrafluoroethylene (PTFE), acrylonitrile butadiene styrene plastic (ABS), and Q235 circular steel. These four materials were chosen because they represented distinct levels of support stiffness. The five internal support conditions and the measured elastic moduli and densities are shown in Figure 2. All the tube fillers were rods with a diameter of 25 mm and a length of 95 mm. A bonding method was not used between the outer wall of the support and the inner wall of the 304 thin-walled tubes as well as the ends of the plug heads.

### 2.2. Loading Method

(a) Loading equipment: Under the condition of a loading rate below 120 mm/min, the Shenzhen Wance 100-ton HUT106D microcomputer-controlled electrohydraulic servo universal testing machine (*v*_max_ = 120 mm/min, located at the Army Engineering University of PLA in Nanjing, China) was used. For loading rates above 120 mm/min, the w+b servohydraulic dynamic fatigue testing system (*v*_max_ = 3000 mm/min, located at Southeast University in Nanjing, China) was employed.
(b) Loading rate: Five levels of loading rates were set: 2, 3, 15, 75, and 120 mm/min. The fracture matrix of the specimen was obtained based on these five support conditions. To explore the critical loading rate for the switching of fracture mode under different support conditions, a loading rate within the range of 0.6–3000 mm/min was set for some specimens. A quasistatic strain rate in the order of 1.05 × 10^−4^−5.26 × 10^−1^ s^−1^ was achieved.
(c) Sampling frequency: When the loading rate was below 120 mm/min, the sampling frequency was 10 Hz. When the loading rate was above 120 mm/min, the sampling frequency was 200 Hz.

### 2.3. Testing Procedure

(a) Calibration of the testing machine: Through a series of elastic tensile tests on the circular steel specimens, it was found that the inherent displacement and the tensile force of the testing machine exhibited a good linear relation, which satisfied repeatability. During data processing, the displacement of the testing machine caused by the tensile force was deducted. After the above calibration process, the displacement value became closer to the true extension of the specimen.
(b) The specimens and fixtures were cleaned and degreased before stretching to prevent them from slipping off during the stretching process.
(c) The specimen was installed and then stretched until it broke according to the set loading rate.
(d) The test data were recorded and then processed using the Origin data processing software.

## 3. Results and Analysis

### 3.1. Effect of Internal Support on the Fracture Mode of 304 SSTWTs

**Figure 3:**
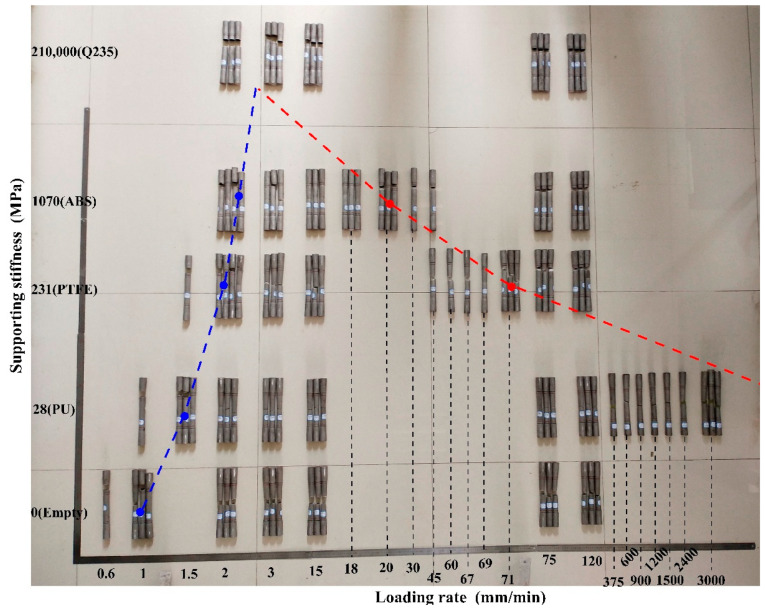
Fracture macromorphology of standard specimens of 304 SSTWTs.

Through failure tests of 110 effective specimens, the fracture mode matrix of 304 SSTWTs under different tube fillers and loading rates was obtained, as shown in Figure 3. It is clear that the tube filler stiffness strongly affects the fracture mode of these tubes. One mode corresponds to the fracture in the middle of the tube section, and the other mode represents the fracture near the clamping side. As the loading rate increases, the thin-walled tube exhibits upper and lower critical loading rates where the fracture mode switches. In Figure 3, the groups with blue dots (●) and red dots (●) are all critical groups in which some specimens were broken in the middle and others were broken at the ends, and the lower and upper critical loading rates are shown by blue and red dotted lines, respectively (due to the limitation of the ultimate tensile loading rate of the testing machine, the highest loading rate of the specimen was 3000 mm/min, and the upper critical loading rate of PU-filled specimen was not obtained). The triangle formed by the two lines represents the fracture mode of the middle tube section. The outer area of the triangle indicates the fracture mode at the clamping side, and the narrow areas on both sides of the dotted line represent the transitional zones of fracture mode switching.

**Figure 4:**
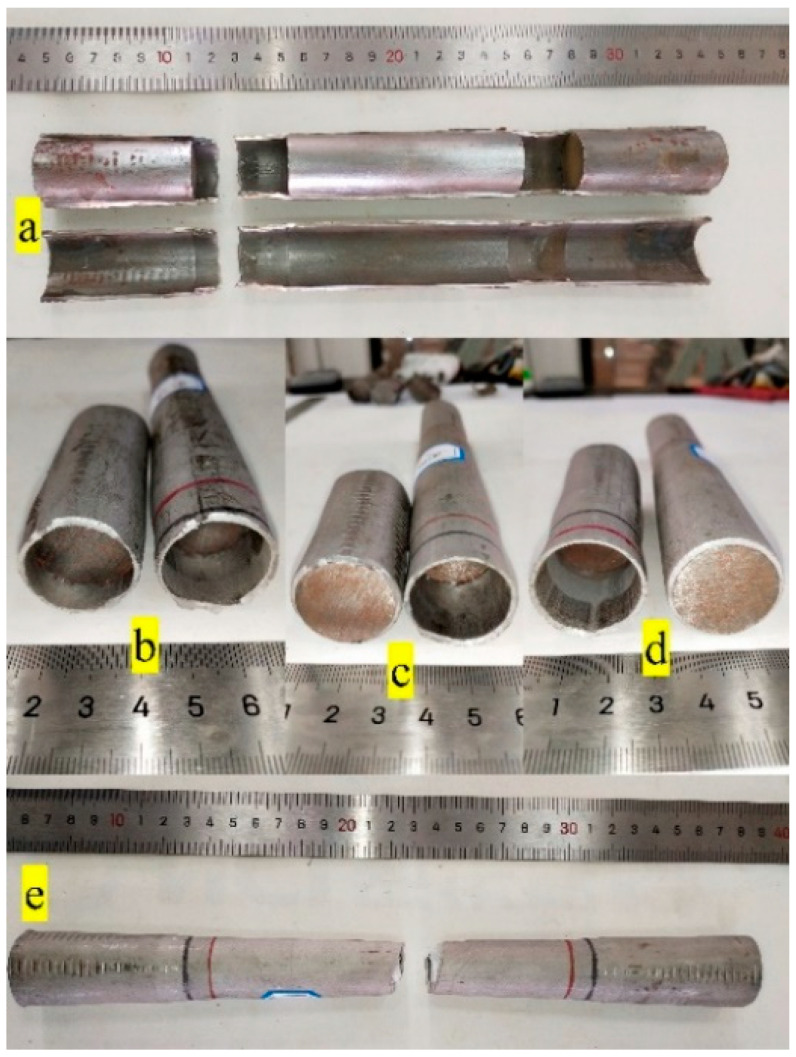
Typical fracture mode. (**a**) Hollow pipe section caused by asynchronous deformation. (**b**–**d**) Clamping-side fracture mode. (**e**) Middle fracture mode.

Under axial tensile load, due to the support effect of the internal filler, the specimen deforms more uniformly. A small section of the empty tube without filling is formed between the support and the plug head, as shown in Figure 4a. In the final stage of deformation, an obvious lateral necking occurs in this section. The necking of this empty tube competes with that of the middle part of the tube section. As the tube filler stiffness increases, the necking of the middle part of the tube section becomes more difficult. When the tube filler stiffness becomes smaller, the necking in the middle part of the tube section is easier.

When the loading rate is smaller than the lower critical loading rate, the lateral necking speed in the empty tube between the support and the plug head is greater than that in the middle of the tube section, and the specimen breaks at the end close to the clamping side. In the test, it is found that there are two types of fractures near the clamping side. One is the fracture in the middle of the end empty tube, as shown in Figure 4b. In this case, the necking tendency in the middle part of the end empty tube is dominant. The other is the fracture at the flange of the plug head or support, as shown in Figure 4c,d. In this case, the shear stress at the flange dominates, and a combined tensile–shear failure occurs at the flange.

When the loading rate is larger than the upper critical loading rate, the lateral necking speed in the empty tube between the support and the plug head is larger than that in the middle of the tube section, which causes the specimen to break near the clamping side, and the fracture mode is the same as that shown in Figure 4b–d.

When the loading rate is between the upper and lower critical loading rates, the lateral necking speed in the middle of the tube section is larger than that at the small section of the empty tube. The specimen breaks in the middle of the entire specimen, as shown in Figure 4e.

At critical loading rates, the fracture mode of tubes is uncertain. If failure occurs on the clamping side, the mechanical properties of the tube under the same working conditions are lower than those in the case when failure occurs in the middle of the tube section. Hence, tube failure should occur in the middle part of the tube section. The end of the tube can be properly reinforced to avoid the corresponding failure mode in projects.

**Figure 5:**
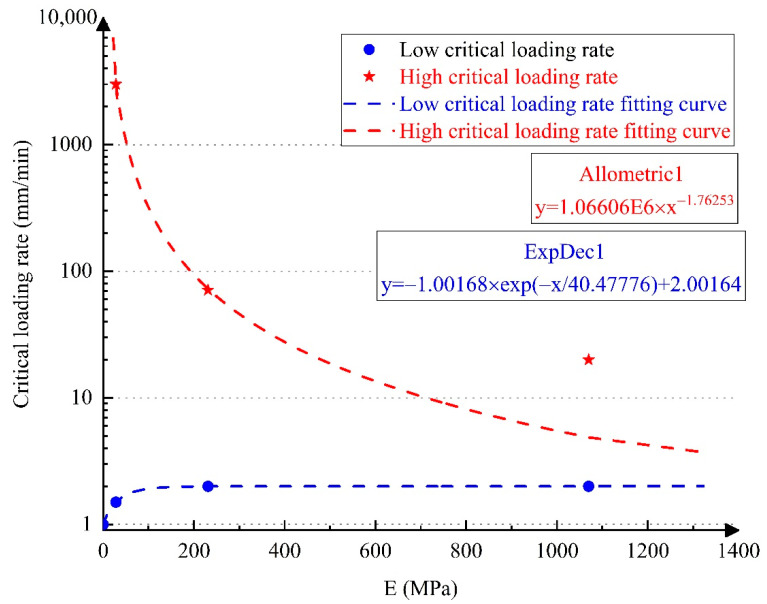
Fitting curves of critical loading rate. Note: the R-square values of the upper and lower critical loading rates are 0.99992 and 0.99998, respectively.

Considering the elastic modulus of the tube filler as an independent variable, the upper and lower critical loading rates of the thin-walled tube where the fracture mode switches under different working conditions can be treated as dependent variables. Allometric1 and ExpDec1 functions were used to fit the upper and lower critical loading rates, as shown in Figure 5.

It is evident in Figure 5 that when the tube filler stiffness is lower than 200 MPa, the critical loading rate is more sensitive to the change in the tube filler stiffness. As the tube filler stiffness decreases, the upper critical loading rate rises rapidly, while the lower critical loading rate decreases significantly. When the tube filler stiffness is higher than 200 MPa, the critical loading rate is not so sensitive to the variations in the tube filler stiffness. Under flexible internal support conditions, the applicable loading rate range of the middle fracture mode is rather wide, and the rigid support causes the specimen to easily break at the end.

The fitting curves of the upper and lower critical loading rates intersect at a point on the right side, and the tube filler stiffness at the intersection point is approximately 1.77 GPa. When the elastic modulus of tube filler is greater than the abscissa value of the intersection, the fracture of 304 SSTWTs can only occur near the clamping side. When the elastic modulus of the tube filler is smaller than the abscissa value of the intersection, the tubes can have two types of failure modes, i.e., clamping-side fracture mode and middle fracture mode of the tube section. 

### 3.2. Failure Test Results of Standard Specimens of 304 SSTWTs

To investigate the influence of internal support on the mechanical properties of 304 SSTWTs, the mechanical properties of the tubes under five support conditions (empty, PU, PTFE, ABS, and Q235 circular steel) and different loading rates were obtained based on the failure test of 110 specimens, and the results are shown in Figure 3. The relative increases in the UNTS, extension, and fracture energy of the specimen with tube fillers with respect to those of the empty tube specimen are analyzed. The statistical results are listed in Table 2, Table 3 and Table 4, in which the values in red represent negative increment, and the values in purple, green, and blue represent the maximum increment in the middle fracture mode when the tube fillers are PU, PTFE, and ABS, respectively.

### 3.3. Effect of Internal Support on the Mechanical Properties of 304 SSTWTs

**Figure 6:**
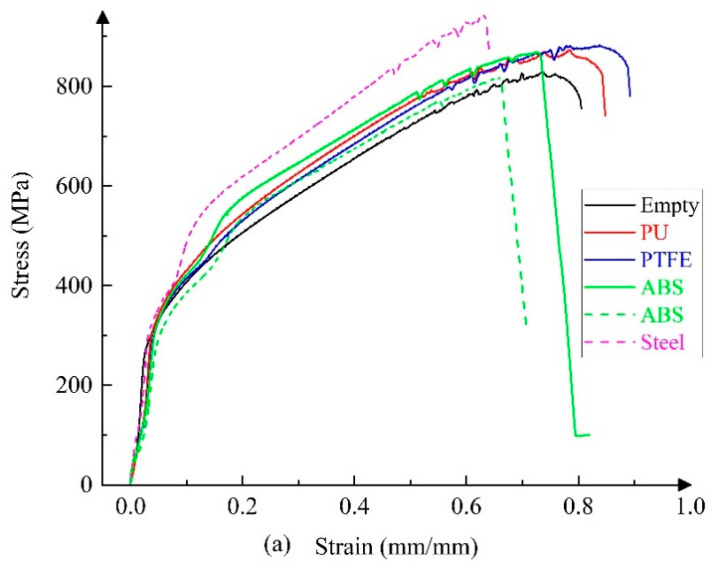

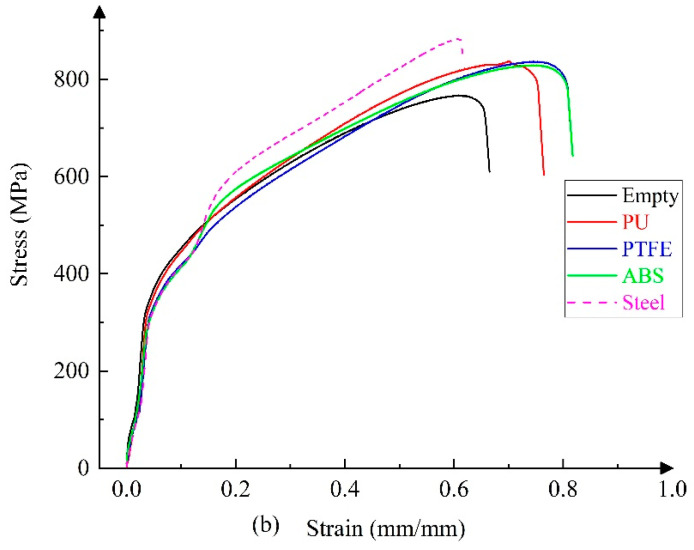
Nominal stress–strain curves under five support conditions with loading rates of 3 mm/min (**a**) and 15 mm/min (**b**).

The displacement–load curves of the specimen under uniaxial tension were obtained through experiments, and the nominal stress–strain curves of the specimen were calculated. Figure 6 shows typical nominal stress–strain curves under different working conditions with loading rates of 3 mm/min and 15 mm/min. Here, the solid line represents the middle fracture mode of the tube section, and the dotted line indicates the fracture mode near the clamping side.

It is evident in Figure 6 that the effect of the internal support has a strong impact on the ultimate strength and extension of 304 thin-walled tubes under each loading rate. In the middle fracture mode of the tube section, the effect of internal support positively affects the ultimate strength and the extension of the tube (except for the case of ABS tube filler with a loading rate of 3 mm/min). In the clamping-side fracture mode, the effect of internal support has a negative impact on the ultimate strength at low loading rates and has a positive impact at a high loading rate. The effect of internal support primarily has a negative effect on the tube extension under the clamping-side fracture mode. As the tube filler stiffness increases, the ultimate strength exhibits an overall increasing trend, and the extension initially increases and then decreases.

**Figure 7:**
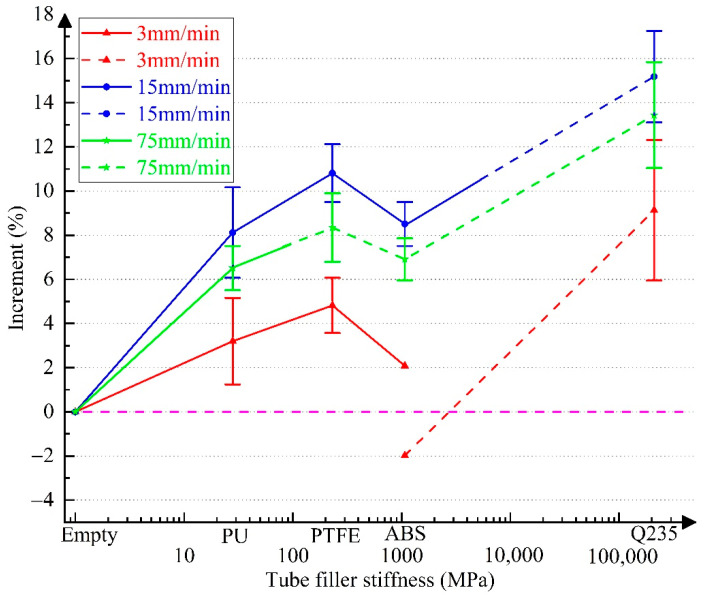
Variation in the increment of ultimate strength as a function of tube filler stiffness. Note: The solid and dotted lines represent the middle fracture mode and the one-side fracture mode, respectively.

**Figure 8:**
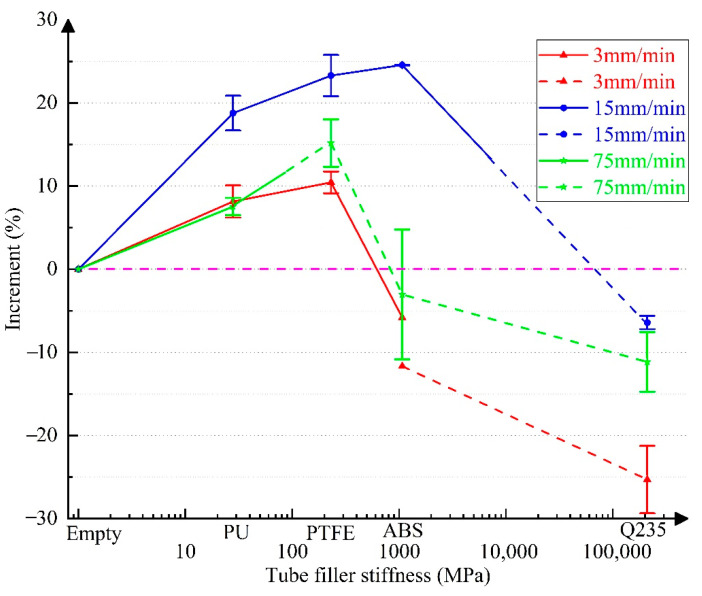
Variation in the increment of extension as a function of tube filler stiffness. Note: the solid and dotted lines represent the middle fracture mode and the one-side fracture mode, respectively.

**Figure 9:**
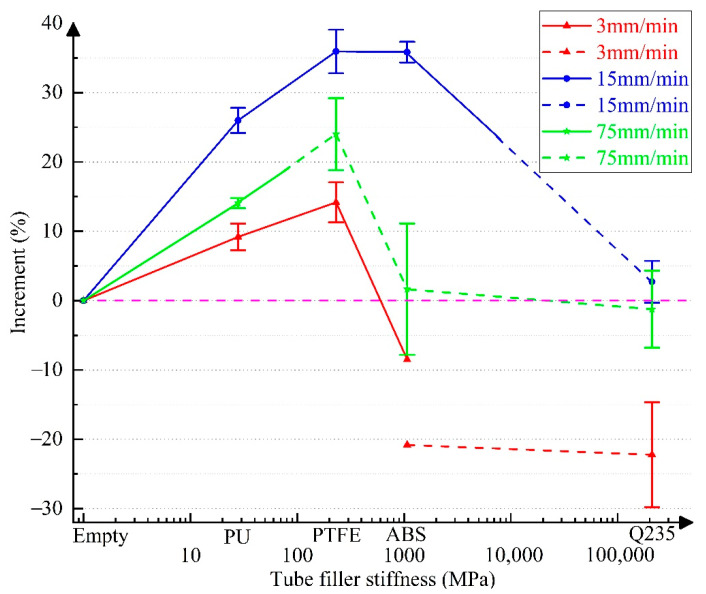
Variation in the increment of fracture energy as a function of tube filler stiffness. Note: the solid and dotted lines represent the middle fracture mode and the one-side fracture mode, respectively.

For quantitatively analyzing the influence of tube filler stiffness on the increment in the mechanical properties of 304 SSTWTs, according to the test data in Table 2, Table 3 and Table 4, the incremental curves of ultimate strength, extension, and fracture energy of specimens with different tube fillers relative to the empty tube are shown in Figure 7, Figure 8 and Figure 9. Each increase in data in Figure 7, Figure 8 and Figure 9 was derived from the measurement results of at least three specimens, and the corresponding standard deviations are shown in Table 2, Table 3 and Table 4. Here, the solid line represents the middle fracture mode of the tube section, and the dotted line indicates the fracture mode near the clamping side.

The following inferences can be drawn from Figure 7, Figure 8 and Figure 9:

(a) As the tube filler stiffness increases, the ultimate strength initially increases and then decreases. After the fracture mode switches, the ultimate strength is still higher than that of the empty tube.
(b) As the tube filler stiffness increases, both extension and fracture energy increase first and then decrease.
(c) When the tube filler stiffness reaches ~500–1000 MPa, the tube extension begins to demonstrate a negative increment. As the tube filler stiffness further increases, the decreasing trend becomes more obvious. Therefore, the rising trend of fracture energy is also reversed.
(d) When the loading rate is 15 mm/min, the increment in the mechanical properties of the tube becomes maximum. In the middle fracture mode of the tube section, the ultimate strength, extension, and fracture energy exhibit a maximum increment of 10.81% (PTFE), 24.56% (ABS), and 35.94%. (PTFE), respectively.

In the case of internal rigid support (Q235 circular steel), all the fracture modes correspond to the clamping-side fracture. The UNTS, extension, and fracture energy increase from 5.51% (2 mm/min) to 15.18% (15 mm/min), −25.3% (3 mm/min) to −6.41% (15 mm/min), and −22.23% (3 mm/min) to 2.73% (15 mm/min), respectively. The test results show that a rigid support can significantly improve the ultimate capacity of tubular specimens, but it has a strong undesired effect on the extension rate, which leads to a significant reduction in the fracture energy. This can seriously affect the fracture toughness of tubular specimens under ultimate working conditions.

When the tube filler is a deformable material, the tube is more likely to fracture on the clamping side with the increase in the elastic modulus of support. For example, when the tube filler is ABS (*E* = 1070 MPa), the applicable loading rate range for the middle fracture mode is only 2–20 mm/min. When the tube filler is PU (*E* = 28 MPa), the applicable loading rate range for the middle fracture mode is greater than 1.5 mm/min.

When the loading rate is 15 mm/min, all the supporting conditions exhibit optimal performance. The physical and mechanical properties of the four supporting materials and the maximum increment in the mechanical properties under different working conditions are listed in Table 5.

It can be seen from Table 5 that:

(a) ABS and PTFE cause the most significant improvement in the mechanical performance of 304 SSTWTs at the ideal loading rate (15 mm/min). The increment in the fracture energy exceeds 35%. Tubular specimens filled with PU under the same loading rate have a slightly inferior performance, where the fracture energy increases by 26.01%.
(b) From the perspective of the applicability of loading rates, when the supporting material is ABS and PTFE with a larger elastic modulus, the tube is prone to fracture on the clamping side under higher and lower loading rates. This causes relatively large uncertainty in the mechanical properties of tubular specimens. When the supporting material is PU with a smaller elastic modulus, the tube tends to fracture on the clamping side only under extremely low loading rate conditions.
(c) It is noteworthy that PTFE has a large density and high cost. ABS has the smallest density, but it is expensive. PU has a lower density, which is only ½ of the PTFE density, and the unit volume price is about 1/3 of that of PTFE and ABS. In addition, PU exhibits strong deformability with a wide elastic range. Thus, it does not break due to excessive deformation and can maintain the integrity of failed tubular specimens.

To summarize, among the three flexible tube fillers, PU has the best overall performance.

## 4. Mechanism of Enhancement Effect of Internal Support

### 4.1. Stress State Analysis of the CFST Specimen

**Figure 10:**
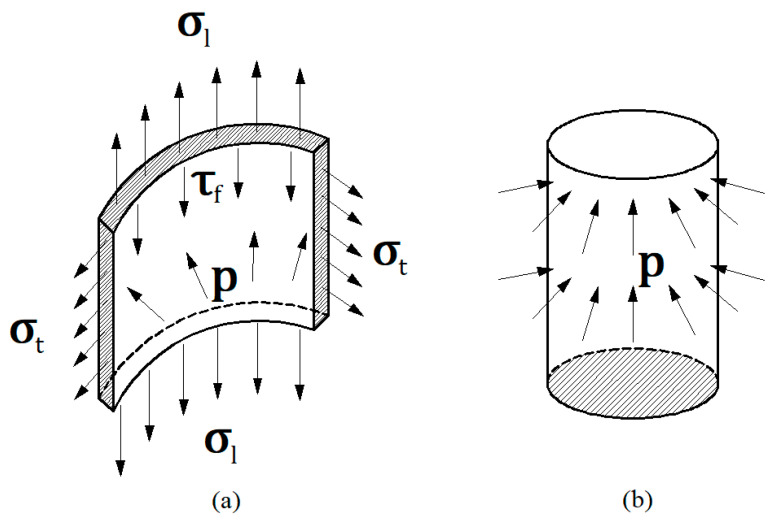
Structural stress state analysis of a concrete-filled steel tube (CFST).

Earlier studies have demonstrated that the increase in the ultimate tensile strength of a CFST specimen relative to the hollow steel tube is mainly due to the change in the stress state of the steel tube [32,33,34,35,36,37,38]. As shown in Figure 10, the empty tube is in a unidirectional tensile stress state under the axial load. The CFST specimen produces a longitudinal tensile stress $\sigma_{l}$ under axial tension, and the “clamping” characteristics of the outer tube generate a tensile stress $\sigma_{t}$ in the transverse direction of the steel tube. Furthermore, the “supporting” effect of the core concrete generates a contact pressure stress *p* between the steel tube and the concrete. For the tube that satisfies the Mises yield condition, an increase in hoop stress can enhance the ultimate tensile strength of the CFST to a certain extent [32].

### 4.2. Stress States of 304 SSTWTs under Different Internal Supports

**Figure 11:**
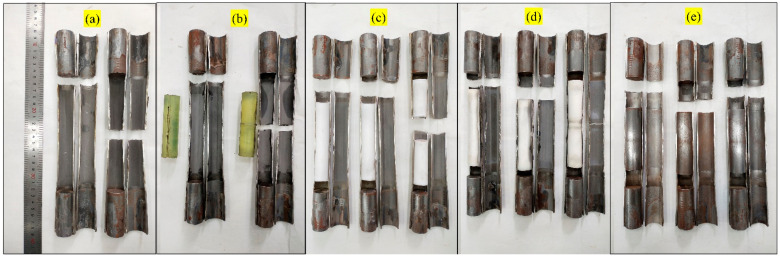
Longitudinal section of the fractured specimen. (**a**) Longitudinal cross-sectional view of empty tube test specimen. (**b**–**e**) The longitudinal cross-sectional views of the specimens when the tube fillers are PU, PTFE, ABS and Q235 respectively.

**Figure 12:**
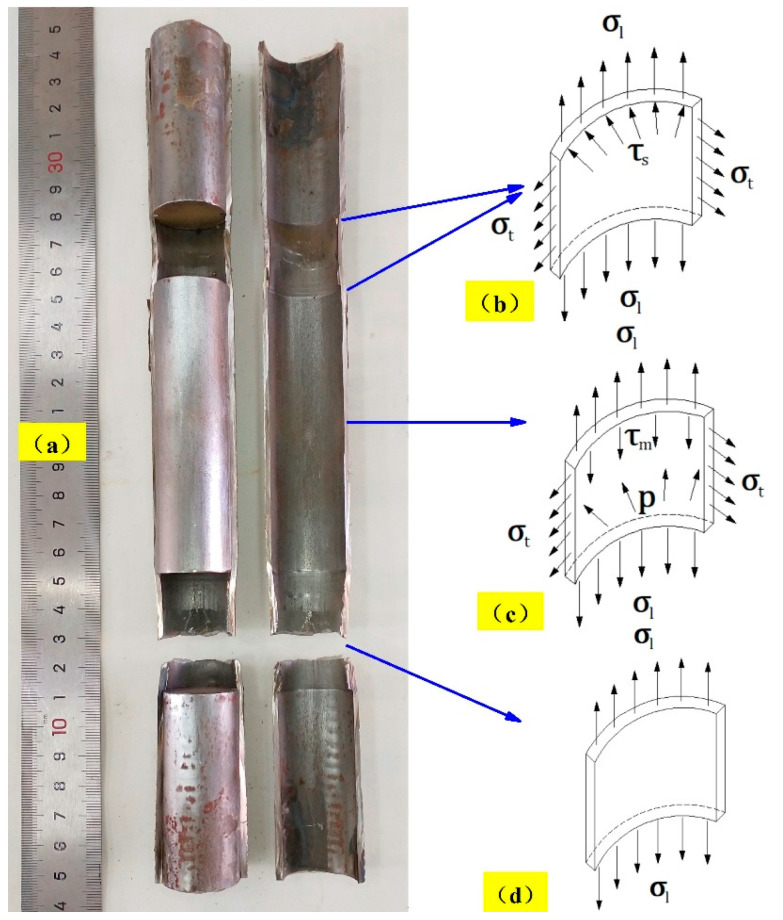
Stress analysis of a 304 SSTWT. (**a**) Longitudinal cross-sectional view of filled specimen. (**b**) Bidirectional tension and lateral shear stress state. (**c**) Axial and hoop tension, radial compression, and longitudinal shear stress state. (**d**) Unidirectional tension stress state.

In this study, the fractured specimens under the five working conditions were longitudinally cut, and the macroscopic morphology after the cut is shown in Figure 11. Under axial loading, 304 SSTWTs exhibit three typical stress states, as presented in Figure 12. At the flanges of the rigid plug head located at the end and the support, the circular tube is in a bidirectional tension ($\sigma_{l}$ and $\sigma_{t}$) and lateral shear ($\tau_{s}$) stress state, as shown in Figure 12b. In the middle part of the tube section, the stress state of the outer tube is similar to that of the CFST structure. The circular tube is in axial and hoop tension ($\sigma_{l}$ and $\sigma_{t}$), radial compression (*p*), and longitudinal shear ($\tau_{m}$) stress state, as shown in Figure 12c. In the middle part of the empty tube between the plug head and support, the circular tube is in a unidirectional tension ($\sigma_{l}$) stress state, as shown in Figure 12d.

The three typical stress states have a mutual competing relation, which is closely related to the loading rate and elastic modulus of the tube filler. The critical loading rate curves in Figure 3 and the corresponding fitting equation shown in Figure 5 can be applied for the determination of this competitive relation. In the two-dimensional space composed of the loading rate and elastic modulus, when (v,E) is within the upper and lower critical loading rates, the stress state shown in Figure 12c dominates. During the loading process, due to the interaction between the steel tube and the tube filler and the necking trend in the middle of the tube section, the contact compressive stress *p* and the longitudinal shear stress $\tau_{m}$ rise with the increase in the longitudinal strain. The longitudinal shear stress facilitates the transmission of tensile force from the steel tube to the tube filler to a certain extent, thereby contributing to an improvement in the ultimate tensile strength. The flexible tube filler has a prohibitive effect on the clamping of the tube section, which can slow down the rapid necking in the middle part of the steel tube. Thus, the entire tube section is more uniformly deformed. Consequently, it fully takes advantage of the mechanical properties of each material and increases the extension rate of the steel tube. The failure mode corresponds to Figure 4e. When (v,E) is outside the triangular area enclosed by upper and lower critical loading rates, the stress states shown in Figure 12b,d are dominant. The circular tubes have combined tensile–shear failure or uniaxial tensile fracture, and the failure mode corresponds to Figure 4b–d. Under this condition, the tube section is not fully deformed, and the specimen fractures near the clamping side in advance. Thus, its mechanical properties are not utilized fully, and the mechanical indicators may have a negative increment.

When the filling material is Q235 circular steel, since the tube filler stiffness is extremely large, it is equivalent to reducing the parallel length of the empty tube of the specimen when the empty tube between the plug head and the support breaks in this case. The increase in the ultimate tensile strength of the specimen filled with Q235 circular steel may be primarily attributed to the size effect.

### 4.3. Working Mechanism

**Figure 13:**
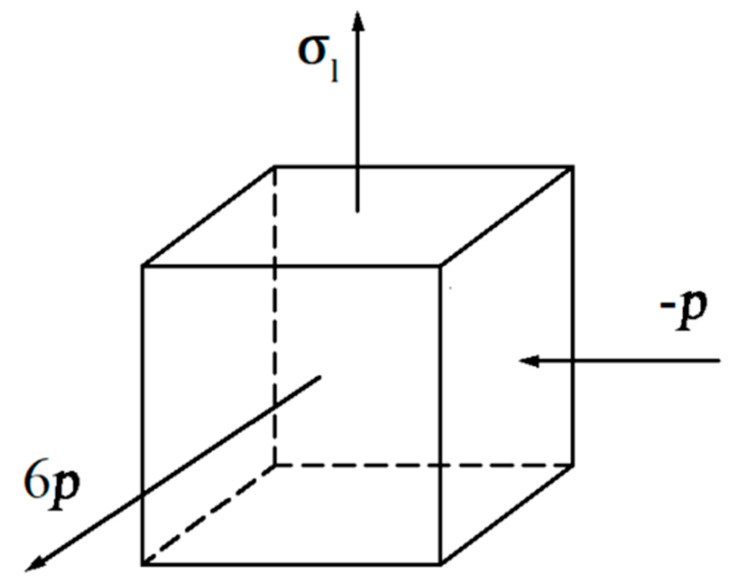
Stress state of a 304 SSTWT in the middle of the tube section.

The following assumptions are considered:

(a) Since $\tau_{m}$ is much smaller than $\sigma_{l}$, $\sigma_{t}$, and *p*, the longitudinal shear stress $\tau_{m}$ in the middle of the tube section is ignored.
(b) $\sigma_{t}$ is constant in the thickness direction, so $\sigma_{t}$ and *p* satisfy the following relation: $\sigma_{t}=\frac{pd_{0}}{2\delta }$. Substituting the specimen size in this test yields $\sigma_{t}=6p$ (assuming that $d_{0}/\delta$ does not change during the stretching process). With internal support, the stress state of a 304 SSTWT in the middle of the tube section is shown in Figure 13.
(c) The second invariant $J_{2}$ of the deviator stress under the fracture of a 304 SSTWT is constant.

When the empty tube is stretched to break, i.e., p=0,

(1) $$J_{2}=\frac{\sigma_{HU}^{2}}{3}$$

When fracture occurs under the condition of internal support, i.e., p>0,

(2) $$J_{2}=\frac{1}{6}[{\left(\sigma_{lU}-6p\right)}^{2}+{\left(6p+p\right)}^{2}+{\left(-p-\sigma_{lU}\right)}^{2}]=\frac{1}{3}(\sigma_{lU}^{2}-5\sigma_{lU}p+43p^{2})$$

By solving Equations (1) and (2) simultaneously, it can be obtained that

(3) $$\sigma_{lU}^{2}-5\sigma_{lU}p+43p^{2}=\sigma_{HU}^{2}$$

According to Equation (3),

(4) $${\left(\frac{\sigma_{lU}}{\sigma_{HU}}\right)}^{2}-5\frac{\sigma_{lU}}{\sigma_{HU}}\frac{p}{\sigma_{HU}}+43{\left(\frac{p}{\sigma_{HU}}\right)}^{2}=1$$

**Figure 14:**
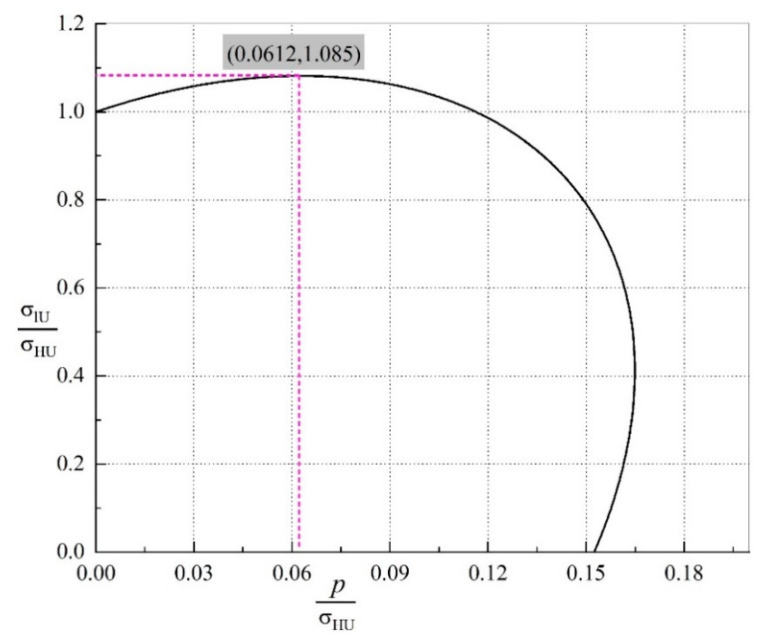
Variation trajectory of ultimate fracture strength with *p*.

According to Equation (4), the variation in the ultimate fracture strength $\frac{\sigma_{lU}}{\sigma_{HU}}$ as a function of $\frac{p}{\sigma_{HU}}$ is examined. The corresponding trajectory under the breaking of a 304 SSTWT is shown in Figure 14. Since *p* becomes larger with the increase in the tube filler stiffness, the relation between $\sigma_{lU}$ and *E* can be qualitatively described, as shown in Figure 14.

According to Figure 14, the following inferences can be drawn:

(a) When $0.0612\sigma_{HU}>p>0$, as *p* increases, the maximum increment of $\sigma_{lU}$ reaches 8.5%, i.e., when the elastic modulus of tube filler is less than a certain critical value, $\sigma_{lU}$ rises with the increase in *E*.
(b) A large value of tube filler stiffness is not always favorable. When the tube filler stiffness becomes greater than a certain critical value (i.e., when *p* exceeds the critical value), as *E* increases, $\sigma_{lU}$ exhibits a decreasing trend. This also explains the observations in Figure 7, i.e., why the elastic modulus of ABS is greater than that of PTFE, whereas the improvement in ultimate tensile strength by ABS is less than that by FTFE.

## 5. Discussion

The existing studies on the effect of internal support mainly focus on the experimental determination of the tensile properties of CFSTs. However, the impact of flexible support on the mechanical properties of the tubes has been rarely investigated. In this study, the effect of internal support on the tube under different loading rates was experimentally investigated. The results revealed that the flexible support is more beneficial than the rigid support for improving the ultimate tensile properties of the tubes. The ultimate strength, ductility, and fracture energy of the tubes were significantly improved, and the structural toughness of the specimens was optimized.

### 5.1. Comparative Analysis of the Effect of Internal Support on the Mechanical Properties of Tubes

Related studies [32,33,34,35,36,37,38] have demonstrated that tube filler under a specific loading rate can improve the tensile strength and the rigidity of tubes. Nevertheless, the ductility and fracture energy of tubes were not analyzed in these studies. Table 6 compares the test conditions and the increment in the mechanical properties between these references and the present study. The mechanical properties of tubes with PU as the support material are also summarized.

Comparative analysis of PU and concrete:

(a) According to the tensile properties of tubular specimens in Refs. [32,33,34,35,36,37] (Ref. [38] proposed a double-layer CFST sandwich structure, which is different from Refs. [32,33,34,35,36,37]), the filling of concrete in a hollow steel tube can increase the ultimate bearing capacity by 5–11%. The present results indicate that the filling of PU can improve the ultimate bearing capacity of tubes by 2.85–8.12%. Basically, the flexible support can provide the equivalent improvement as a rigid support.
(b) The typical PU flexible support can significantly improve the ultimate deformability of tubular specimens while increasing the ultimate bearing capacity. At a loading rate of 15 mm/min, the tube extension can be increased by 1.73–18.78% if PU is used as the filling material. The variation in the extension rate of CFST has not been clarified in the existing literature. However, from the perspective of the fracture mode of a rigid support (Q235 circular steel) specimen, if the end is not reinforced, the tube is prone to fracture on the clamping side. Therefore, the extension rate is significantly reduced, which is not conducive to the safety of components and structures.
(c) When PU is used as the filling material, the fracture energy of tubes can be increased by 5.55–26.01%. When a rigid support (Q235 circular steel) is used, the increment in the fracture energy of tubes is −22.23–2.73%. The increase in the ultimate bearing capacity can barely compensate for the adverse effect on the extension rate at the optimal loading rate (under 15 mm/min). In most cases, the fracture energy is considerably reduced. Generally, the elastic modulus of concrete is 25–40 GPa, and the deformability is weak. Its effect is close to that of the rigid support, i.e., it has a negative effect on the toughness of tubular specimens.
(d) From the perspective of weight, the density of PU is less than half of the concrete density, which is more conducive for the lightweight design of structures. In terms of construction technology, the use of PU for filling is convenient and does not require maintenance, and it is also suitable for assembled steel–tube composite structures. On the contrary, a CFST structure requires a longer maintenance period, complicated processing and manufacturing, stricter requirements for site and equipment, and higher overall cost.

Overall, the “tube + flexible tube filler” structure proposed in this study can significantly improve the ultimate mechanical properties and fracture toughness of tubes. This structure is simple, lightweight, inexpensive, and exhibits high strength and ductility. Furthermore, it is quite suitable for prefabricated steel–tube composite structures.

### 5.2. Comparative Analysis of the Effect of Internal Support on the Fracture Mode of Tubes

**Figure 15:**
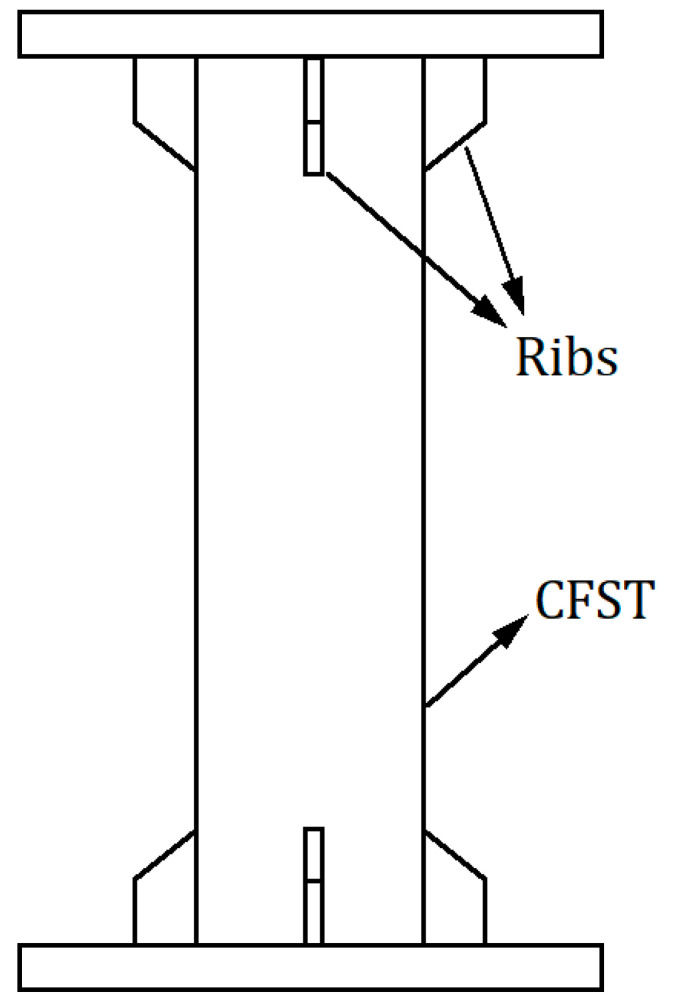
Schematic diagram of a CFST member with reinforced ribs.

Earlier studies [32,33,34,35,36,37,38] only focused on tensile strength, bending rigidity, and eccentric loading. For analyzing the tensile properties of CFSTs, they did not consider the fracture mode of the tubes. In CFST tensile tests, ribs were welded at the end of specimens (Figure 15) for reinforcement. When the tube is reinforced at the end, the fracture of specimens can only occur near the middle region. Consequently, it is difficult to examine the influence of internal support on the fracture mode of tubes (the weld zone is fractured because it is more likely to exhibit brittle characteristics than other areas, and the switching of the fracture mode is not caused by the effect of internal support).

In this study, the specimen end was not reinforced. The effect of internal support on the fracture mode of tubes was explored (the typical fracture mode is shown in Figure 4), and the upper and lower critical loading rates of 304 SSTWTs under different support rigidities were obtained.

The condition in which the ends of 304 SSTWTs with a preset tube filler are reinforced is examined as follows. In actual projects, the ends of tensioned tubular components are mostly reinforced. If the end of the tube with the preset tube filler is reinforced with ribs (Figure 15), tube failure can only occur near the middle of the tube section. In such a case, the end fracture mode cannot have an adverse impact on the tensile properties. According to Figure 7, Figure 8 and Figure 9, it can be predicted that when the end is reinforced with ribs, flexible tube fillers such as PTFE may be more ideal for improving the ultimate tensile properties of 304 SSTWTs.

### 5.3. Application and Future Directions

Owing to the supporting effect of the internal material, the rapid lateral necking of the circular tube during stretching was restricted. The bearing capacity of the circular tube increased. Meanwhile, its ductility and fracture energy enhanced significantly. The combination of these two makes up for the disadvantages of the mechanical properties of tension tubes effectively. With an extremely small increase in cost and weight, the potential of the mechanical properties of tubes was fully utilized, and an effective structural form was established, which has broad application prospects in engineering.

However, the proposed approach has the following limitations, which require further investigation:

(a) In this experiment, only one type of 304 stainless steel tube was tested, and the effects of tube material, length–diameter ratio, and –diameter ratio on the effect of internal support were not considered.
(b) This test focuses on the impact of internal support on the tensile properties and fracture mode of tubes. The improvement in the mechanical properties of tubes under complex stress conditions such as bending, tension, torsion, and impacts needs to be further investigated.
(c) Through the comparison of four supporting materials, it is concluded that PU has the best overall performance, but it is relatively expensive. Finding inexpensive tube fillers with a suitable elastic modulus, further reducing the manufacturing cost and structural weight, and the industrialization of internal support structures are worth exploring.

## 6. Conclusions

In this paper, the ultimate tensile properties and fracture mode of 304 SSTWTs in the case of tube fillers with different elastic moduli were experimentally investigated. The main results of the study can be summarized as follows:

(1) Due to the supporting effect of internal filling materials, the lateral necking of circular tubes toward the axis of symmetry during stretching was restricted. The ultimate tensile bearing capacity, extension, and fracture energy of the specimens were significantly increased. The flexible tube filler and the thin-walled tension tube formed an efficient composite structure. When the loading rate was 15 mm/min and the fracture corresponded to the middle fracture mode, the maximum increments in the ultimate tensile strength, extension, and fracture energy of tubes were 10.81% (PTFE), 24.56% (ABS), and 35.94% (PTFE), respectively.
(2) Two fracture modes were obtained: the middle fracture mode and the clamping-side fracture mode. Under the middle fracture mode, internal support had a positive effect on the ultimate strength and extension of tubes (only one case showed reduction). Under the clamping-side fracture mode, the effect of internal support had a negative effect on the ultimate strength under low loading rates and a positive effect under high loading rates. The effect on the tube extension was primarily negative.
(3) Different internally filled specimens exhibited upper and lower critical loading rates where the fracture mode switched. The upper critical loading rate was more sensitive to the variations in the tube filler stiffness, whereas the lower critical loading rate was insensitive to these variations. When the loading rate was between the upper and lower critical loading rates, the failure of the composite structure corresponded to the middle fracture mode, which facilitated an improvement in the ultimate tensile properties of composite structures. Under flexible internal support, the applicable loading rate of the middle fracture mode had a wide range. As the tube filler stiffness increased, the range of loading rate gradually decreased. When the elastic modulus of the tube filler was greater than a certain value (estimated to be 1.77 GPa), the 304 SSTWTs could only be fractured near the clamping side.
(4) Given the increase in the mechanical properties and the fracture mode, flexible support was found to be better than rigid support for improving the ultimate tensile properties of tubes to a certain extent. Among the three flexible tube fillers tested, PU exhibited the best overall performance. Using PU as the tube filler, the UNTS, extension, and fracture energy of the tube at a loading rate of 15 mm/min were enhanced by 8.12%, 18.78%, and 26.01%, respectively, without significantly affecting the weight and cost. Additionally, the applicable range of loading rate was wide.

Compared to bulky CFSTs, the flexible tube filler facilitates convenient, lightweight, and inexpensive construction. In particular, it is suitable for prefabricated steel–tube composite structures. Overall, this study provides a novel route for improving the comprehensive performance of steel–tube composite structures under ultimate loading conditions, which is extremely important to enhance the safety of structural design and save construction costs.

## Figures and Tables

**Table 1:** Chemical composition of a 304 stainless steel thin-walled tube (SSTWT).

Chemical Composition (%)						
C	Si	Mn	P	S	Ni	Cr
0.065	0.54	1.27	0.033	0.019	8.02	17.32

**Table 2:** Ultimate nominal tensile strength (UNTS) of the failure test for standard specimens of 304 SSTWTs.

Tube Filler		Material	Empty	PU	PTFE		ABS		Q235	Nominal Strain Rate (/s)
		*E* (MPa)	/	28	231		1070		210,000	
	Loading Rate (mm/min)	Fracture Mode	Middle	Middle	Middle	One-Side	Middle	One-Side	One-Side	
UNTS (Mpa) UNTS = *N*/S_0_	2	Average	876.81	901.80	915.47	864.12	908.92	853.10	925.11	3.51 × 10^−4^
		STD	12.72	31.80	/	/	/	/	5.60	
		Increment	/	2.85%	4.41%	−1.45%	3.66%	−2.70%	5.51%	
	3	Average	836.10	862.83	876.42	/	853.45	819.57	912.42	5.26 × 10^−4^
		STD	8.88	16.40	10.42	/	/	/	26.57	
		Increment	/	3.20%	4.82%	/	2.08%	−1.98%	9.13%	
	15	Average	766.11	828.31	848.94	/	831.30	/	882.44	2.63 × 10^−3^
		STD	4.89	15.68	10.07	/	7.67	/	15.86	
		Increment	/	8.12%	10.81%	/	8.51%	/	15.18%	
	75	Average	750.32	799.16	/	812.95	/	802.19	851.15	1.32 × 10^−2^
		STD	3.43	7.52	/	11.62	/	7.13	17.99	
		Increment	/	6.51%	/	8.35%	/	6.91%	13.44%	
	120	Average	744.39	799.28	/	785.26	/	806.89	814.44	2.11 × 10^−2^
		STD	16.98	13.81	/	6.60	/	10.19	13.75	
		Increment	/	7.37%	/	5.49%	/	8.40%	9.41%	
Loading rate range of the middle fracture modes (mm/min)			1–??	1.5–X X > 3000	2–71		2–20		None	/

The values in red represent negative increment, and the values in purple, green, and blue represent the maximum increment in the middle fracture mode when the tube fillers are PU, PTFE, and ABS, respectively.

**Table 3:** Extension of failure test for standard specimens of 304 SSTWTs.

Tube Filler		Material	Empty	PU	PTFE		ABS		Q235	Nominal Strain Rate (/s)
		*E* ( MPa)	/	28	231		1070		210,000	
	Loading Rate (mm/min)	Fracture Mode	Middle	Middle	Middle	One-Side	Middle	One-Side	One-Side	
Extension (mm)	2	Average	77.52	78.86	84.87	71.56	79.98	67.74	58.45	3.51 × 10^−4^
		STD	1.60	3.28	/	/	/	/	2.28	
		Increment	/	1.73%	9.48%	−7.69%	3.17%	−12.62%	−24.60%	
	3	Average	76.05	82.25	83.99	/	71.64	67.18	56.81	5.26 × 10^−4^
		STD	0.80	1.48	1.00	/	/	/	3.09	
		Increment	/	8.15%	10.44%	/	−5.80%	−11.66%	−25.30%	
	15	Average	62.37	74.08	76.90	/	77.69	/	58.37	2.63 × 10^−3^
		STD	0.66	1.31	1.54	/	0.03	/	0.50	
		Increment	/	18.78%	23.30%	/	24.56%	/	−6.41%	
	75	Average	63.23	68.00	/	72.82	/	61.31	56.18	1.32 × 10^−2^
		STD	1.73	0.66	/	1.81	/	4.94	2.27	
		Increment	/	7.54%	/	15.17%	/	−3.04%	−11.15%	
	120	Average	66.53	67.73	/	69.86	/	58.48	53.05	2.11 × 10^−2^
		STD	2.29	0.41	/	0.35	/	1.89	3.58	
		Increment	/	1.80%	/	5.01%	/	−12.10%	−20.26%	
Loading rate range of the middle fracture modes (mm/min)			1–??	1.5–X X > 3000	2–71		2–20		None	/

**Table 4:** Fracture energy of failure test for standard specimens of 304 SSTWTs.

Tube Filler		Material	Empty	PU	PTFE		ABS		Q235	Nominal Strain Rate (/s)
		*E* (MPa)	/	28	231		1070		210,000	
	Loading Rate (mm/min)	Fracture Mode	Middle	Middle	Middle	One-Side	Middle	One-Side	One-Side	
Fractureenergy (J)	2	Average	9853.54	10,400.80	11,275.25	8580.00	10,529.71	7988.67	7673.98	3.51 × 10^−4^
		STD	136.69	641.50	/	/	/	/	247.48	
		Increment	/	5.55%	14.43%	−12.92%	6.86%	−18.93%	−22.12%	
	3	Average	9413.00	10,277.59	10,748.17	/	8614.44	7456.34	7320.25	5.26 × 10^−4^
		STD	96.72	182.34	270.66	/	/	/	712.04	
		Increment	/	9.19%	14.18%	/	−8.48%	−20.79%	−22.23%	
	15	Average	7246.40	9131.48	9850.81	/	9843.59	/	7444.44	2.63 × 10^−3^
		STD	128.03	129.52	228.22	/	108.34	/	218.35	
		Increment	/	26.01%	35.94%	/	35.84%	/	2.73%	
	75	Average	7351.28	8384.58	/	9116.61	/	7472.98	7260.53	1.32 × 10^−2^
		STD	191.45	53.46	/	381.64	/	695.83	409.33	
		Increment	/	14.06%	/	24.01%	/	1.66%	−1.23%	
	120	Average	7616.14	8372.25	/	8378.41	/	7298.99	6579.16	2.11 × 10^−2^
		STD	330.57	123.74	/	187.63	/	387.48	528.08	
		Increment	/	9.93%	/	10.01%	/	−4.16%	−13.62%	
Loading rate range of the middle fracture modes (mm/min)			1–??	1.5–X X > 3000	2–71		2–20		None	/

**Table 5:** Comparative analysis of the mechanical and physical performance for four kinds of tube fillers.

Material	*E* (MPa)	ρ (kg/m^3^)	$v_{m}$ (mm/min)	Maximum Increment (%)			Comprehensive Assessment
				UNTS (Middle)	Extension (Middle)	Fracture Energy (Middle)	
Q235	210,000	7867	None	/	/	/	Worst
ABS	1070	1044	2–20	8.51	24.56	35.84	Third
PTFE	231	2170	2–71	10.81	23.30	35.94	Second
PU	28	1103	1.5–X X > 3000	8.12	18.78	26.01	Best

**Table 6:** Comparative analysis of the effect of support on the increment in the mechanical properties of steel tubes.

Authors	Ref.	Structure	ρ (kg/m^3^)	*E* (MPa)	Loading Rate (mm/min)	Mechanical Property	Maximum Increment (%)	Note
Han et al.	[32]	CFST	2200–2500	25,000–40,000	0.6	Ultimate tensile strength	About 11	
Zhou et al.	[33]	SCFST			2	Ultimate tensile strength	5.2	*
Ye et al.	[36]	CFSST			0.6	Ultimate tensile strength	5–10	Concentric tension
Pan et al.	[37]	CFST			/	Yield strength	10	
Li et al.	[38]	CFDST			5	Ultimate tensile load	20.8	Concentric tension
Gao et al.	This study	PU	1103	28	3	Ultimate tensile strength	3.20	Middle fracture modes
						Extension	8.15	
						Fracture energy	9.19	
					15	Ultimate tensile strength	8.12	
						Extension	18.78	
						Fracture energy	26.01	

Note: (a) * The loading rate was not mentioned in the original paper. After contacting the corresponding author, it was confirmed that the loading rate was ~2 mm/min. (b) Refs. [32,33,34,35,36,37,38] did not involve the study of the ductility and fracture energy of CFST specimens.

## Data Availability

The data presented in this study are available on request from the corresponding author.

## Notations

$D_{0}$	Original external diameter of tube (mm)
$d_{0}$	Original internal diameter of tube (mm)
δ	Wall thickness of tube (mm)
$S_{0}$	Original cross-sectional area (mm^2^)
$L_{0}$	Original gauge length (mm)
$L_{c}$	Parallel length (mm)
$L_{h}$	Clamping length (mm)
$L_{t}$	Total length of test piece (mm)
$L_{b}$	Length of plug head (mm)
E	Elastic modulus (MPa)
ρ	Density of filler (kg/m^3^)
*N*	Axial tensile force (N)
SSTWT	Stainless steel thin-walled tube
UNTS	Ultimate nominal tensile strength
CFST	Concrete-filled steel tubes
CFDST	Concrete-filled double-skin steel tube
SCFST	Squared concrete-filled steel tubes
CCFST	Circular concrete-filled steel tube
CFSST	Concrete-filled stainless steel tube
STD	Standard deviation
$\sigma_{l}$	Longitudinal tensile stress (MPa)
$\sigma_{t}$	Transverse tensile stress (MPa)
$\sigma_{HU}$	Ultimate tensile breaking strength of hollow tube (MPa)
$\sigma_{lU}$	Ultimate longitudinal fracture stress (MPa)
p	Contact compressive stress (MPa)
τ	Longitudinal shear stress (MPa)
$\tau_{s}$	Transverse shear stress on the gripping side (MPa)
$\tau_{m}$	Longitudinal shear stress in the middle of the tube (MPa)
v	Loading rate (mm/min)
$v_{m}$	Loading rate range of middle fracture modes (mm/min)
$J_{2}$	Second invariant of deviatoric stress
